# Gender Differences in Kinlessness and Loneliness: Psychological Resilience as a Moderated Mediator

**DOI:** 10.1093/geroni/igaf122.1131

**Published:** 2025-12-31

**Authors:** Jianan Li, Sarah Sunghye Kang, Shekhar Chauhan, Lené Levy-Storms

**Affiliations:** University of California, Los Angeles, Los Angeles, California, United States; University of California, Los Angeles, Los Angeles, California, United States; Florida State University, Tallahassee, Florida, United States; UCLA Luskin School of Public Affairs/Social Welfare, Los Angeles, California, United States

## Abstract

Being kinless risks loneliness in later life due to having fewer close, familial relationships, but less is clear about how this relationship manifests across kinlessness types. Psychological resilience (PR) may buffer against loneliness, offsetting risks associated with kinlessness. Given the long-established relationship differences by gender, the effects of kinlessness, loneliness, and PR may vary among men and women, yet this process remains understudied. Using the 2016 and 2018 Health and Retirement Study, this study examined (1) how PR mediates the relationship between kinlessness types (having a partner and children, having children but no partner, having a partner but no children, having no partner and no children) and loneliness in later life and (2) how this mediated relationship is further moderated by gender (N = 9,384). Adjusted linear regression showed adults without kin had the highest loneliness, followed by those with children but no partner and those with a partner but no children. PR significantly mediated the relationship between kinlessness and loneliness: adults with no kin and those with children but no partner showed lower PR, leading to higher loneliness. This pathway was further moderated by gender: the association between having no kin and loneliness was nearly three times stronger for males than for females. Gender also moderated the relationship between kinlessness and loneliness: while females had similar loneliness trends and levels across kinlessness types, males with no kin experienced the highest levels of loneliness. This study highlights the critical role of a partner in PR and loneliness, especially for males.

